# Microalgae-Based Biostimulants: Effects on Growth and Stress Resistance in Agricultural Crops

**DOI:** 10.3390/plants14223488

**Published:** 2025-11-15

**Authors:** Carla Arenas Colarte, Iván Balic, Óscar Díaz, Ignacio Cortes, Adrián A. Moreno, Maximiliano J. Amenabar, Miguel Castro Retamal, Nelson Caro Fuentes

**Affiliations:** 1 Centro de Investigación Austral Biotech, Facultad de Ciencias, Universidad Santo Tomás, Santiago 8320000, Chile; carenas8@santotomas.cl (C.A.C.); icortes8@santotomas.cl (I.C.); mamenabar@santotomas.cl (M.J.A.); miguelcastro@santotomas.cl (M.C.R.); 2 Departamento de Acuicultura y Recursos Agroalimentarios, Área Prioritaria de Investigación (API3), Programa Fitogen, Universidad de Los Lagos, Osorno 5311157, Chile; ivan.balic@ulagos.cl (I.B.); oscar.diaz@ulagos.cl (Ó.D.); 3 Centro de Biotecnología Vegetal, Facultad de Ciencias de la Vida, Universidad Andres Bello, Santiago 8370146, Chile; adrian.moreno@unab.cl; 4 Escuela de Biotecnología, Facultad de Ciencias, Universidad Santo Tomás, Santiago 8320000, Chile; 5 Laboratorio de Microbiología Aplicada, Departamento de Ciencias Básicas, Facultad de Ciencias, Universidad Santo Tomás, Santiago 8320000, Chile

**Keywords:** microalgae-based biostimulants, abiotic stress, sustainable agriculture, plant growth

## Abstract

Microalgae grow rapidly, require minimal space, can proliferate in non-agricultural land, do not compete with human food sources, and can be cultivated in a variety of environments, including wastewater. They are considered an ecological source of bioactive compounds, offering an environmentally friendly alternative to conventional industrial production methods, which are often resource-intensive. It is important to emphasize that both the species of microalgae and the specific culture conditions play a decisive role in the generation and storage of valuable bioactive compounds, which can act as biostimulants. Biostimulants are organic compounds or microorganisms capable of enhancing crop quality parameters by optimizing nutrient and water use efficiency, while also strengthening tolerance to abiotic stress. The aim of this article is to provide an updated understanding of biostimulants, their modes of action, and their role in regulating plant responses to abiotic stress. It further incorporates examples of successful trials that demonstrate the advantageous applications of microalgae-based biostimulants, while also addressing the barriers and limitations to their commercialization and integration into sustainable agricultural practices.

## 1. Introduction

Urban expansion and climate change are reducing the amount of arable land available for agriculture worldwide, thereby threatening the global food supply, which must double by 2050 [1]. To meet the growing demand for food, industrial agricultural practices have been widely adopted, including the use of synthetic fertilizers, pesticides, and growth promoters aimed at increasing crop productivity. However, the continuous application of these chemical compounds has deteriorated soil quality, particularly by altering its physicochemical profile and associated microflora, ultimately leading to a decline in crop yields [2]. The excessive use of pesticides and fertilizers to boost agricultural production has also exerted adverse environmental impacts, as it has increased the accumulation of chemical residues that pose risks to human, animal, and plant health, including toxic effects on the biosphere [3]. This occurs because much of these chemical products are released directly into fields, thereby disrupting microbial communities and contaminating water reserves. Consequently, it is imperative to introduce changes in current agricultural practices to reduce or replace the excessive use of pesticides and fertilizers [4]. The disposal of synthetic fertilizers into the soil may result in land acidification, depletion of organic matter and humus, and loss of beneficial soil organisms, all of which negatively affect plant development [5]. With respect to water pollution, high nitrogen (N) inputs applied to crops may leach into groundwater, causing eutrophication. This process can eliminate aquatic fauna, promote the proliferation of invasive species, and reduce recreational opportunities due to unpleasant odors and water contamination [6]. In addition to these severe environmental consequences associated with their application, the production of these chemicals also contributes to atmospheric pollution through the release of toxic gases (e.g., N_2_O, NH_4_^+^, CO_2_, CH_4_) [7]. To address these issues, biotechnological solutions have been proposed as promising strategies to enhance crop yields, given their potential to transform agricultural systems and mitigate emerging challenges [2]. Biostimulants (BS) have emerged as an effective strategy to address the growing demand for alternative products, supported by their bioactivity, absence of toxic effects on non-target organisms, and low ecological persistence. The use of biological and renewable products that enhance plant growth through diverse mechanisms has already become a consolidated practice across a variety of agricultural crops [8]. Recently, the European Biostimulants Industry Council (EBIC) defined “plant biostimulants as substances or microorganisms that, when applied to plants or the rhizosphere, stimulate natural processes to improve nutrient uptake efficiency, tolerance to abiotic stress, and crop quality, regardless of the nutrient content” [9]. Biostimulants differ from synthetic fertilizers in that they elicit cellular and adaptive responses without relying on the presence of minerals in their formulations [10]. Thus, the concept of “biostimulant” refers to its function rather than its chemical composition. For this reason, biostimulants, biocontrol agents, and biofertilizers should be carefully distinguished [11]. Biostimulants encompass a broad range of substances and microorganisms that, when applied at low concentrations to plants or soils, modulate physiological and biochemical processes to enhance plant growth, nutrient-use efficiency, and tolerance to abiotic stress. In contrast to biofertilizers, which contain living microorganisms primarily intended to increase the availability or supply of essential nutrients, biostimulants do not serve as a direct source of nutrients. Instead, they stimulate the plant’s intrinsic metabolic responses, thereby improving crop performance and reducing dependence on conventional chemical fertilizers [12]. Plant biostimulants are usually applied via soil irrigation or foliar spraying at low concentrations, which distinguishes their mode of action from inorganic synthetic fertilizers [13]. They are classified according to their origin or active ingredients into three categories: microbial, non-microbial, and residue derived [14]. Microbial biostimulants include plant growth-promoting microorganisms naturally present in the rhizosphere. Non-microbial and residue-derived biostimulants consist of organic compounds obtained from a wide variety of sources, including underutilized biomass from the food and agricultural industries, thereby contributing to sustainable agriculture [15]. Organic sources encompass a broad set of compounds, with the most common being protein hydrolysates, amino acids, humic acids, biopolymers, seaweed extracts, and living microorganisms such as bacteria, yeasts, fungi, and microalgae. In contrast, inorganic sources include beneficial chemical elements such as trace minerals or inorganic salts, for example, phosphite or silicon salts [16].

### Importance of Biostimulants in Modern Agriculture

**Figure 1:**
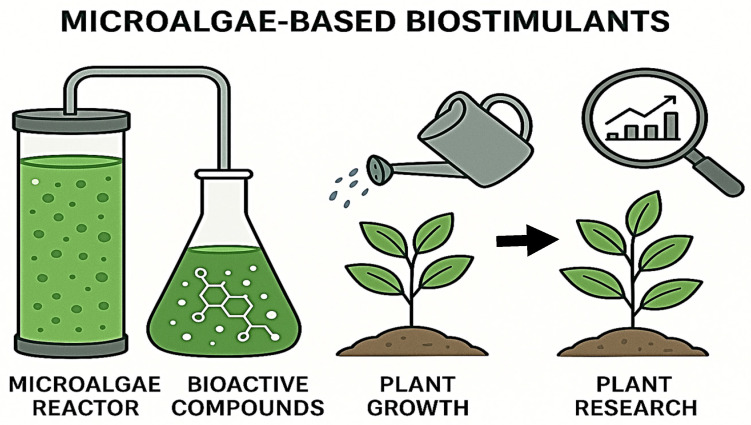
**Schematic representation of microalgae-based biostimulants in agriculture.** Figure illustrates the conceptual framework of microalgae-based biostimulants for agricultural applications. The scheme outlines the process from microalgae cultivation in bioreactors to the extraction of bioactive compounds, their application to plants, and the subsequent evaluation of plant responses. This visual representation highlights the role of microalgae as a sustainable source of bioactive molecules that enhance plant growth, improve tolerance to abiotic stress, and promote antioxidant activity, providing an eco-friendly alternative to conventional agricultural inputs.

Significant progress has been made in the application of biostimulants within sustainable agricultural practices. These innovative products can enhance plant development, nutrient uptake, yield and crop quality, stress tolerance, and soil fertility, while collectively reducing the environmental impact of agriculture. Although still an emerging market, the biostimulant sector demonstrates considerable potential, which explains the growing interest of both the scientific community and agribusiness stakeholders in exploring new sources of plant biostimulants [17]. Recently, biostimulants containing living microorganisms have attracted notable attention from industry and academia, as they are considered particularly effective in optimizing plant growth and development under field conditions [18]. Furthermore, diverse techniques are employed for their application, including inoculants, hydrolysates, and extracts [17] (Figure 1). Within this context, microalgae have emerged as environmentally friendly biostimulant/biofertilizer candidates capable of improving both crop quality and yield [19].

Microalgae have garnered substantial interest in both the scientific community and the biostimulant industry, primarily due to their ability to thrive on arable land while producing high biomass yields year-round. Moreover, new cultivation strategies employing wastewater and CO_2_ enable the large-scale production of biomass at affordable costs, while simultaneously incorporating perspectives of local and circular economies [20]. Microalgae have been shown to exert biostimulant effects by promoting higher yields, higher germination rates, better root development and better fruit quality. Additionally, metabolites derived from microalgae have been reported to improve soil fertility, confer resistance to abiotic stress, stimulate defensive responses against pathogens and infections, and optimize the uptake of soil nutrients such as nitrogen (N), potassium (K), phosphorus (P), and essential minerals [21].

## 2. Microalgae as a Source of Biostimulants

### 2.1. Main Genera and Species Used

Despite the significant potential benefits of microalgae in agriculture, the selection of suitable and promising strains remains a considerable challenge. The choice of high-potential microalgal strains is determined by several criteria. One of the most relevant aspects is achieving rapid and uniform growth and productivity, along with a simple cultivation system (growth in nutrient media and the use of waste resources) to generate sufficient biomass for crop trials. Strains with a high cell growth rate are recommended due to their relatively short doubling time [22]. It is also crucial to adopt new production methods that reduce excessive water consumption and minimize the energy footprint. In this context, strategies such as microalgal biorefineries and circular economy models can be combined to address these challenges. A microalgal biorefinery can be defined as a process that enables the integral use of algal biomass after its production. This approach is grounded in the sustainability of microalgal cultivation by coupling biomass production with the recovery of high-value industrial molecules [23]. Metabolites and extracts derived from species such as *Scenedesmus* spp., *Spirulina platensis*, *Chlorella* spp., *Dunaliella* spp., *Calothrix elenkini*, *Acutodesmus* spp., among others, have been reported [24,25]. For instance, *Chlamydomonas reinhardtii, Dunaliella* sp., *Scenedesmus* spp. and *Chlorella* spp. are widely used due to their high capacity to produce phytohormones that promote the growth of various plants [26]. Beyond increasing metabolite levels, the application of biostimulants has also been shown to enhance germination rates, leading to earlier flowering and a higher number of flowers. Moreover, polysaccharide extracts from microalgae such as *Chlorella sorokiniana*, *Chlamydomonas reinhardtii*, *Porphyridium* spp., and *Dunaliella salina* have been demonstrated to increase the activity of antioxidant enzymes—including superoxide dismutase, peroxidase, and catalase—in tomato plants subjected to salt stress, thereby suggesting their role as effective biostimulants [27]. In recent years, there has been increasing recognition of the potential of Chlamydomonas as a biotechnological tool for bioremediation, biofertilization, biomass production, and bioproducts. Chlamydomonas has established itself as a basis for the generation of various relevant biotechnological products, including several types of biofuels and high-value-added products [28]. Overall, strain selection significantly influences production techniques and technologies, affects the generation of value-added metabolites, and determines the choice of downstream processing methods [29]. In the past decade, interest in green microalgae as a source of compounds with biostimulant activity has grown substantially. Numerous studies have evaluated candidate species, highlighting their potential due to the wide range of properties they offer for agricultural applications. Table 1 summarizes several reports on the use of biostimulants derived from green microalgae and their effects as plant growth promoters.

### 2.2. Bioactive Compounds of Agricultural Interest

Microalgae are currently widely employed in agriculture due to their ability to produce numerous bioactive molecules beneficial to crops, such as phenols, phytohormone-like substances, amino acids, terpenoids, and polysaccharides [35]. This high production of bioactive compounds derives from their capacity to adjust to diverse environmental conditions and adapt to different nutritional modes: mixotrophic (ability to utilize reduced organic carbon and light sources), heterotrophic (fermentative method using organic carbon), and autotrophic (fully photosynthetic) [36]. These chemical compounds are known to activate mechanisms in plants that enhance nutrient uptake, mitigate abiotic stress, and stimulate secondary metabolite pathways [24]. Furthermore, microalgae can establish highly effective symbiotic interactions with soil microorganisms, as they can coexist within the phycosphere and benefit from the joint production of bioactive compounds (such as exopolysaccharides, amino acids, proteins, and vitamins) or from significant activity analogous to cytokinins and/or auxins [37]. For example, *Methylobacterium* spp. have great biotechnological potential in agriculture, as they generate phytohormones, promote plant growth through N_2_ fixation, and offer defense against pathogens and pollutants. It has recently been proposed that indole-3-acetic acid (IAA) generated by algae influences mutualistic interactions between *Chlamydomonas* and *Methylobacterium* spp. When nitrogen is scarce, *Chlamydomonas* produces IAA from L-tryptophan through a process mediated by L-amino acid oxidase (LAO1) [38].

#### 2.2.1. Phytohormones

Phytohormones are low-molecular-weight signaling compounds that are naturally produced in plants and exert stimulatory effects at minimal concentrations in several developmental processes, including root and shoot formation, fertilization, plant senescence, tissue differentiation, defense mechanisms, and tolerance to diverse biotic and abiotic stresses. According to their functions, phytohormones are classified into auxins, cytokinins, gibberellins, abscisic acid (ABA), and ethylene [39]. These molecules are particularly intriguing because, in some cases, they are structurally identical in microalgae and plants and are synthesized through highly conserved biosynthetic pathways [26]. It is also noteworthy that the impact of each phytohormone is not determined solely by its concentration, but also by the relative levels of other phytohormones. This phenomenon results from endocrine communication—that is, the interplay between the nature and proportions of phytohormones—which produces direct phenotypic effects in plants [40]. Under survival conditions, plants undergo alterations in hormonal regulation, generally characterized by increased levels of abscisic acid (ABA) and ethylene (ETH), accompanied by decreased levels of cytokinins and auxins. These signaling pathways are species-specific and variable, enabling microbial biostimulants to act on plant metabolism and enhance stress tolerance. The wide diversity of microbial communication mechanisms has been shown to influence multiple plant response processes, including epigenetic modifications, transcriptional and translational regulation (protein synthesis), phytohormone regulation, and detoxification of reactive oxygen species (ROS) [41,42]. Phytohormones are synthesized at different stages of the microalgal growth curve. For instance, in *Chlorella vulgaris* and *Arthrospira platensis*, the auxin indole-3-acetic acid (IAA) is produced during the stationary phase, whereas total cytokinin levels are highest during the early exponential phase [43].

##### Auxins

Auxins are primarily associated with root growth. They constitute a group of naturally occurring low-molecular-weight molecules in plants that induce growth and are involved in multiple biological processes. Some of the most common auxins include indole-3-acetic acid (IAA), indole-3-acetamide (IAM), 2-phenylacetic acid, 4-chloroindole-3-acetic acid, and indole-3-butyric acid (IBA), with IBA being particularly effective in inducing root formation due to its greater stability [29,44]. Microalgae such as *Chlorella* spp., *Coenochloris* spp., *Acutodesmus* spp., *Scenedesmus* spp., and *Chlorococcum* spp. have been reported to contain auxins at concentrations ranging from 0.18 to 99.83 nmol g^−1^ of dry biomass, primarily consisting of IAA and IAM. Among these, IAA was identified as the predominant auxin in nearly 24 microalgal species [45]. One study assessed auxin activity and the ability of algal extracts to promote root development, revealing that biomass application enhanced root generation by approximately 100% and 240% at concentrations of 0.5 and 2.0 g L^−1^, respectively. At the highest concentration tested, biomass derived from wastewater-grown microalgae exhibited a superior biostimulant effect [46]. Comparable results were observed in *Chlorella vulgaris* cultivated in both freshwater and wastewater. Roots not only provide structural support for plants but also serve as a vital source for absorbing air, water, and nutrients. The application of plant biostimulants derived from microalgae may therefore promote root development, thereby enhancing nutrient uptake, growth, and adaptation to stress conditions [47].

##### Cytokinins

Cytokinins are adenine-derived hormones that regulate plant growth, physiological functions, and responses to abiotic stress [48]. They are mainly produced in root tips, as well as in unripe fruits and seeds. Cytokinins promote lateral shoot development, cytokinesis, and thus cell division [49]. These hormones are also synthesized by other eukaryotic and prokaryotic organisms, including bacteria, fungi, microalgae, and even insects. Importantly, cytokinins can be produced by both pathogenic and beneficial microorganisms, and they have been shown to enhance plant resistance against pathogen infections [50]. Several previous studies have demonstrated that microalgae can act as inducers of pathogen resistance. Extracts from microalgal compounds have been applied in plant–pathogen interactions to trigger resistance responses, and in some cases, the extracts directly inhibited the growth of different pathogens [51,52].

##### Gibberellins

Gibberellins are the key hormones associated with seed germination initiation and stem elongation. Approximately 19 different types of gibberellic acids have been identified in microalgae, with primary functions related to stem elongation, seed germination through enzymatic activation (α-amylase), flowering onset, floral organ development, and reproductive success [45,47]. They also regulate photosynthetic efficiency and promote the redistribution of photosynthates, thereby balancing the source–sink relationship under abiotic stress [53]. Extracts containing gibberellins from *Chlorella vulgaris* have been shown to reduce the negative impacts of heavy metal stress and provide protection against lead and cadmium toxicity [54]. Extracts from *Scenedesmus obliquus* have been reported to stimulate root development and germination rates in common crops [55,56], whereas *Chlorella vulgaris* extracts enhance seedling vigor in beet seeds [57]. Consequently, an effective biostimulant such as the culture supernatant of *C. vulgaris* may directly supply gibberellins and stimulate the biosynthesis of this phytohormone. As a result, the increase in germination index induced by microalgae has been described as a “gibberellin-like effect” [58].

##### Abscisic Acid (ABA)

ABA plays a crucial role in abiotic stress and acts as an important mediator between the soil microbiome and the enhanced ability of plants to withstand water stress. The application of bacteria such as *Azospirillum* and *Pseudomonas* has been shown to promote ABA accumulation in *Arabidopsis thaliana*, tomato, and maize under both laboratory and greenhouse conditions. This, in turn, triggers mechanisms that ultimately reduce water loss in plants [59]. Microorganism-induced ABA accumulation can regulate stomatal closure and chlorophyll production, thereby sustaining photosynthesis and osmotic adjustment. This enhances water-use efficiency and ensures plant survival under drought conditions [60].

#### 2.2.2. Polysaccharides and Exopolysaccharides

Polysaccharides are complex polymeric macromolecules composed of neutral sugars with varying compositions and, consequently, distinct biological functions. These compounds play a key role in stimulating plant growth and metabolic activity by regulating physiological and biochemical processes. Their main effects include promoting root development, improving nutrient availability and mobilization through mineral chelation, and enhancing photosynthesis via increased Rubisco synthesis. Additionally, polysaccharides contribute to tolerance against both biotic and abiotic stresses, functioning as important signaling molecules [61,62]. Among the different microalgal species, cyanobacteria such as *Spirulina platensis*, *Phormidium* spp., *Plectonema* spp., *Calothrix* spp., and *Nostoc* spp. produce polysaccharides that are secreted into the environment and are referred to as exopolysaccharides [63]. Likewise, several microalgae, including *Tetraselmis* spp., *Dunaliella salina*, *Chlorella stigmatophora*, *Chlorella vulgaris*, and *Porphyridium cruentum*, are also capable of synthesizing polysaccharides with biostimulant activity [64].

The application of polysaccharides derived from cyanobacteria and microalgae in the field has demonstrated beneficial effects on host plants. Foliar application of *Spirulina platensis* extracts rich in polysaccharides improved the growth of tomato and pepper plants [65]. Similarly, polysaccharide extracts from *Dunaliella salina*, *Chlorella vulgaris*, *Chlorella sorokiniana*, and *Chlamydomonas reinhardtii* have shown biostimulant properties when applied to tomato seedlings [66]. One study reported that polysaccharides obtained from different microalgae, including *Chlorella*, *Dunaliella*, and *Porphyridium*, act as strong inducers in tomato plants by enhancing the activity of enzymes linked to defense mechanisms and promoting the accumulation of protective metabolites such as polyphenols and steroidal glycoalkaloids [67]. Furthermore, exopolysaccharides produced by *Porphyridium sordidum* were shown to induce the expression of salicylic acid-related genes and phenylalanine ammonia-lyase (PAL) activity in *Arabidopsis thaliana* leaves, leading to a reduced incidence of diseases caused by *Fusarium oxysporum* [68].

#### 2.2.3. Amino Acids and Bioactive Peptides

The use of protein- and amino acid-based biostimulants has been reported to produce numerous beneficial effects in different plant species [69]. Extracts of microalgae containing specific amino acids have been shown to increase protein, pigment, and phytohormone production in plants, which are essential for plant development [70]. Protein hydrolysates and amino acids represent an important category of plant biostimulants and are widely applied in sustainable agricultural practices [71]. Protein hydrolysates consist of short peptides (polypeptides, oligopeptides) and free amino acids, which may also contain small amounts of lipids, polysaccharides, phytohormones, and macro- and micronutrients. These compounds directly regulate carbon and nitrogen metabolism in plants, thereby modulating nutrient uptake and the activity of key enzymes (malate dehydrogenase, citrate synthase, and isocitrate dehydrogenase) within the tricarboxylic acid cycle. Bioactive peptides exert functions like auxins and gibberellins in plants, enhancing overall growth and crop productivity [72].

*Chlorella vulgaris* is one of the few industrial microalgae species capable of heterotrophic growth, which has enabled some of the highest biomass productivities reported for microalgal cultivation. Heterotrophic cultivation has a lower land-use footprint compared to photoautotrophic systems, requires less land and water, and is not dependent on climatic conditions, thereby allowing consistently high cell concentrations. Additionally, this species accumulates high protein levels, ranging from 20% to 64% of its dry weight [73,74]. A biorefinery approach to biomass utilization can also be explored. For example, *Arthrospira platensis* has been used as a protein source, and the residual biomass following protein extraction was evaluated as a plant biostimulant, achieving better results than the complete biomass [75]. Another study reported enhanced plant growth through the use of various amino acids, including glutamine, tryptophan, proline, and methionine, which were found in the biomass of *Tetradesmus obliquus*, suggesting their potential biostimulant role in seed germination and root growth [76]. In addition, biomass derived from *Tetradesmus obliquus* was also shown to be an effective biostimulant for cucumber, mung bean, and garden cress crops [77].

#### 2.2.4. Antioxidant Compounds and Pigments

Antioxidants represent another significant group of nutrients provided by microalgae that enhance crop productivity [45]. Among them, carotenoids are the most relevant, playing a key role in photosynthesis and photoprotection. Carotenoids also influence the coloration of seeds, fruits, and flowers, and serve as precursors of plant signaling hormones such as ABA and strigolactones [78]. The main antioxidant compounds in microalgae include carotenoids such as β-carotene in *Dunaliella salina*, fucoxanthin in *Phaeodactylum tricornutum*, *Isochrysis galbana*, and *Chlorella vulgaris*. Several authors have investigated the carotenoid composition of microalgae in detail. While the role of carotenoids in plant growth and development has been established, the mechanisms of carotenoid conversion and absorption from microalgal biomass remain unclear, highlighting the need for further research [79,80].

The antioxidant properties of these compounds promote seed germination, mitigate the effects of environmental stress factors such as high salinity, drought, and pollutants, increase agricultural productivity, and could reduce the reliance on chemical fertilizers. They achieve this by acting as osmolytes, assisting in heavy metal detoxification, enhancing micronutrient uptake from soil, and optimizing the antioxidant defense enzyme machinery in plant cells [81]. One study reported enhanced growth and chlorophyll content in plants treated with microalgae, associated with more efficient osmotic adjustment. Similar results were obtained with different species; for instance, *C. vulgaris* increased chlorophyll and carotenoid content in the edible parts of lettuce seedlings [82]. Likewise, *Chlorella* showed a consistent effect in *Medicago truncatula*, steadily increasing both chlorophyll and carotenoid levels [31].

## 3. Applications in Agricultural Crops

Microalgae-based biostimulants can be applied as whole cultures, dried biomass, spent medium, supernatants, extracts, or cell suspensions. Thus, the application method varies according to the form of the biostimulant. These modalities can be implemented through different approaches, such as seed priming, seed coating, foliar spraying for leaf surface application, soil drenching by saturating sown soils, or fertigation [31]. These strategies promote soil health and fertility, enhance nutrient availability for plants, stimulate soil microbial activity, support nutrient cycling, and foster plant development [83]. Likewise, they can be employed in hydroponic systems to improve nutrient uptake and plant growth in soilless environments [84].

### Experimental Evidence in Horticultural Crops

As previously mentioned, the application of biostimulants largely depends on the type of crop and its specific requirements, whether for improving nutrient acquisition or enhancing tolerance to biotic and abiotic stresses. Both the method and timing of biostimulant application significantly influence plant responses [82].

To synthesize the main findings reported in the literature regarding the use of microalgae and microorganisms as biostimulants in different plant species, a comparative table is presented below (Table 2). This table highlights the microalgal species evaluated, the model plants used, the type of compound or formulation applied, and the observed effects on growth and developmental parameters. This compilation provides an integrated view of the diversity of organisms tested, the most common application routes, and the importance of factors such as concentration and the type of biomolecules present in the extracts.

Taken together, the studies summarized in Table 2 demonstrate that microalgae and other microorganisms hold considerable potential as biostimulants, either through specific metabolites (phytohormones, polysaccharides, cytokinins) or via the direct use of biomass. However, while these findings show positive effects across different plant species, they also highlight the need to move toward more integrated strategies that consider complex microbial interactions and their applicability on a larger scale.

Research has shown that cross-kingdom and multi-microorganism combinations are more effective than single-strain inoculants. Nevertheless, although microbial consortia have proven to be more efficient, the combination of microorganisms for biostimulant development is not straightforward, nor is it guaranteed to result in additive effects. Consequently, microbial biostimulants may become overly specific, reducing their appeal for large-scale use and commercialization. Still, the functional role of the biostimulant remains the key criterion for selecting which Microorganisms that Promote Plant Growth (PGPMs) are incorporated into formulations. To fully harness their potential, it is essential to develop versatile, broadly applicable formulations capable of meeting diverse agricultural demands [89]. Each field, crop, and production target may require a particular microbial blend, making microbial biostimulants highly specialized products. Variations in composition demand different microbial groups, as well as specific cultivation methods and tailored formulations [90].

## 4. Mechanisms of Action in Plants

The mechanisms underlying the effects of biostimulants on plants remain, in general, poorly understood. The diversity and complexity of the compounds involved have made it difficult to fully elucidate the processes underlying biostimulant activity. Nonetheless, the mechanisms of action of biostimulants related to microorganisms can broadly be divided into two categories: direct and indirect effects. Primary effects include the production of bioactive molecules that enhance nutrient uptake and mitigate stress, while secondary effects involve physiological traits of microorganisms, such as phosphorus solubilization and nitrogen fixation. Although most microalgae lack the enzymatic machinery required for direct nitrogen fixation, they can form symbiotic associations with nitrogen-fixing bacteria. These consortia facilitate the transfer of bioavailable nitrogen to the microalgae, improving growth and productivity while contributing to the internal recycling of nutrients within biotechnological systems [91,92]. The mode of action depends on the nature of the substances present in the biostimulant product. Reported mechanisms of action have been associated with various physicochemical alterations in plants, including reduced lipid peroxidation in membranes, increased chlorophyll content, and enhanced antioxidant activity. Biostimulants can thus be regarded as agents that influence plants either directly or indirectly. Direct effects include the activation of photosynthesis, improved nutrient uptake efficiency, regulation of genetic and metabolic pathways, and modulation of phytohormone release [93,94]. The interaction between a given biostimulant, a particular plant species, and the cultivation conditions also plays a critical role. Moreover, both the plant developmental stage and the environmental conditions at the time of biostimulant application can directly affect treatment success. Biostimulant use directly influences plant growth rate and yield, being especially relevant in leafy vegetables such as lettuce and spinach, as well as in herbs like oregano and basil, and has been shown to support growth under abiotic stress. Biostimulants activate carbon, phosphate and nitrogen metabolism, resulting in enhanced growth, while increasing carbohydrate, leaf, protein, and photosynthetic pigment contents (such as chlorophyll and carotenoids). The application of drought-tolerant phosphate-solubilizing microbes has proven to be a promising strategy to alleviate biotic and abiotic stress [95,96]. To verify whether biostimulants impact plant defense responses, various stress-condition assays are required. These may include nutrient deficiency or excess pollution, high salinity, or water scarcity—the latter being particularly relevant in the current climate context, as noted previously [20]. Although the specific mechanisms underlying stress alleviation by biostimulants are still under investigation, PGPMs share similarities with microalgae in mitigating environmental stress. They achieve this through the production of bioactive metabolites that modulate pathways involved in stress perception and signaling in plants [97].

### 4.1. Induction of Tolerance to Abiotic Stress

Plants have developed significant adaptive strategies to cope with abiotic stress induced by climate change, including de novo synthesis and regulation of phytohormones, accumulation of osmolytes, production of heat shock proteins, and increased activity of enzymatic antioxidants [98]. In addition to promoting plant growth, the application of biostimulants enhances stress resistance through the activation of secondary metabolism. This effect is associated with the increased activity of the PAL enzyme, which is involved in the phenylpropanoid pathway and contributes to the production of phenolics and flavonoids that act as defensive molecules in plants [99].

#### 4.1.1. Drought

Water stress affects plants at biochemical, cellular, and molecular levels. Under drought conditions, stress-associated hormones are activated, osmolytes are produced, stress-protective proteins such as late embryogenesis abundant (LEA) proteins accumulate, and free proline concentration increases as a defensive mechanism [100]. The accumulation of proline in Chlamydomonas is a well-known physiological response to drought or osmotic stress and is commonly regarded as part of the microalgal defense mechanism. However, in this study, we observed that *Methylobacterium* can utilize L-proline as a nitrogen source, thereby establishing a mutualistic exchange in which the bacterium provides ammonium that supports microalgal growth, while the microalgae releases glycerol as a carbon source for the bacterium. These findings suggest that a typically defensive response, such as proline accumulation, may also facilitate beneficial symbiotic interactions under specific environmental conditions, acting as a potential link between stress defense and mutualistic associations [38].

Strategies to cope with water stress include the development of a strong root system, deposition of waxes on the epidermis, shedding of older leaves, regulation of stomatal closure to minimize water loss, alterations in photosynthetic performance, reduced cell proliferation, and the promotion of senescence [101]. Phytohormones and signaling molecules play a crucial role in positively regulating defense mechanisms that plants employ against abiotic stress. For instance, water stress in plants induces an increase in ABA levels, which in turn promotes stomatal closure and regulates plant water balance [102]. Another mechanism for counteracting drought and salt stress is the accumulation of osmolytes such as sucrose, glycine betaine, proline, and trehalose. The increased concentration of these molecules generates a negative osmotic potential within the cell, facilitating water influx to maintain turgor pressure and preserve cellular water balance and osmolarity [103].

#### 4.1.2. Salinity

Salt stress impairs plant development due to chlorophyll degradation and low osmotic potential, leading to stomatal closure, reduced CO_2_ fixation, and enhanced photorespiration and reactive oxygen species (ROS) production [81]. Plants can perceive changes in turgor pressure, subtle variations in intracellular solute concentrations, and mechanical impacts on cellular structures caused by osmotic stress. Calcium signaling has been proposed to play a central role in osmoregulation, as cytosolic free calcium levels in plants increase rapidly and transiently within seconds after exposure to osmotic stress [104]. Salinity tolerance is conferred through various molecular and physiological mechanisms. Genetic modification techniques in plants enable the targeted manipulation of specific traits related to crop quality and survival. Genome-editing tools have proven to be among the most innovative and effective approaches for performing rapid and precise modifications in genes associated with ion transport, thereby regulating osmotic adjustment under salt stress to protect plants from various types of stress and enhance crop yield [105]. In recent years, biostimulants have emerged as a sustainable alternative to mitigate the effects of salt stress. Various extracts from microalgae, fungi, and plant growth-promoting rhizobacteria (PGPR) have been shown to enhance ionic homeostasis, promote the accumulation of osmoprotectants, and activate antioxidant enzymes, thereby reducing oxidative damage and maintaining photosynthetic performance under saline conditions [106,107]. Thus, biostimulants act by modulating plant signaling and defense pathways, complementing or reinforcing endogenous mechanisms of salinity tolerance.

#### 4.1.3. Extreme Temperatures

The reproductive phase of plants is highly vulnerable to heat stress. Stress events during flowering and seed development negatively affect fertility and both yield quality and quantity [108]. To mitigate these effects, plants employ adaptive mechanisms such as heat shock proteins, which stabilize and refold denatured proteins, as well as antioxidant defenses, osmoprotectants, and hormone-mediated responses involving ABA [109]. Despite the substantial economic and social impacts of heat stress on agriculture, current knowledge regarding the role of microalgal biostimulants in alleviating thermal stress in plants remains limited [110]. The scarcity of studies exploring the use of microalgal biostimulants to mitigate heat stress represents a significant research gap. Given the critical importance of this abiotic stress and its anticipated impact on global food security, further in-depth investigations are urgently needed to enhance both scientific understanding and the practical application of microalgae-based biostimulants [111].

Several studies have demonstrated that microalgal extracts and other biostimulants contribute to enhancing plant tolerance under abiotic stress conditions. Table 3 presents recent examples highlighting the positive effects of these compounds on crops exposed to drought, salinity, and other adverse environmental conditions.

The use of biostimulants has been shown to significantly enhance a plant’s ability to withstand abiotic stress, providing a viable option to mitigate the adverse impacts of climate change on agriculture and ensuring yield stability. Biostimulants assist plants in adapting to stressful conditions, thereby facilitating survival or delaying the onset of critical stress periods. Stress-associated metabolites can be found in biostimulants derived from plant extracts or microbial cultures and may possess the ability to “prime” plants for enhanced resistance [3]. Consequently, determining the optimal timing of biostimulant application is as crucial as selecting the appropriate dosage, in order to avoid product losses, increased production costs, and unintended effects. Biostimulants can be applied through foliar spraying, directly to roots at planting to protect seedlings during early growth stages, in nutrient solutions within drift systems, or during flowering and fruit development [116]. Research has shown that combinations of kingdoms and microorganisms are more effective than single-strain inoculants. Studies show synergistic effects of microalgae-bacteria consortia in specific applications (biofertilization, bioremediation). In other contexts, mixing inoculants does not always produce additive or synergistic results due to competitive interactions, ecological incompatibilities, or environmental conditions that limit joint activity. As a result, microbial biostimulants can become overly specific, reducing their attractiveness for large-scale use and commercialization. However, the function of the biostimulant remains the primary factor determining which PGPMs are incorporated into a formulation. To maximize their potential, it is essential to develop versatile and universally applicable formulations that address the diverse needs of the agricultural sector [89].

## 5. Perspectives

The reviewed literature clearly demonstrates that microalgae possess remarkable properties that promote plant growth. However, their widespread use has not yet achieved commercial viability. Major challenges associated with their application in the agrochemical sector include the high cultivation costs, variability in biomass productivity, and the considerable energy and processing demands required to obtain bioactive metabolites with proven efficacy. In this context, a biorefinery approach that enables the integral valorization of microalgal biomass, combined with a circular economy framework that promotes reuse, recycling, and reconditioning with a lower energy footprint, could represent an effective strategy to advance the commercial feasibility of microalgae-based agrochemicals. Due to their high photosynthetic efficiency, rapid growth rates, and ability to accumulate valuable metabolites, microalgae offer important production advantages over other biomass sources. Their cultivation, integrated with nutrient and material recycling, represents a significant opportunity within a regenerative economic system focused on the sustainable exploitation of renewable resources. In this model, biomass generation is coupled with downstream processing to obtain high-value products, while residual fractions can be reused for agricultural applications, thereby closing material and energy cycles [117].

The challenges associated with transitioning from laboratory to field conditions remain critical, as many strains or consortia with exceptional in vitro performance show reduced efficacy under real agricultural conditions [118]. In addition, the lack of unified regulatory frameworks complicates the classification, evaluation, and commercialization of biostimulants, generating uncertainty for both producers and end users. From a practical standpoint, farmer adoption remains limited due to the scarcity of field performance data, perceived economic risks, and the limited availability of standardized products. Furthermore, long-term scientific evidence supporting their efficacy remains insufficient. Most available studies have been conducted under controlled or short-term conditions, limiting our understanding of cumulative and long-term sustainability effects. Therefore, large-scale comparative trials are needed to validate the efficacy, safety, and cost-effectiveness of biostimulants under real agricultural conditions.

In this regard, increasing efforts are being directed not only toward regulation but also toward the standardization of parameters throughout the entire production process. A growing number of stakeholders, including policymakers and experts, now recognize the urgency of establishing scientific principles to substantiate claims regarding plant biostimulants [11]. Indeed, the market for microalgae-derived biostimulants is expanding, with projections estimating a value of USD 13.3 billion by 2025 and USD 32.9 billion by 2035, corresponding to a compound annual growth rate of 9.5% over the forecast period. The integration of biostimulants into organic farming, government initiatives supporting sustainable agriculture, and expanding research on algae-based products are shaping a promising future for the sector. Likewise, the growing application of microalgae in controlled environments, including vertical farming and hydroponic systems, is generating new commercial opportunities [119].

## 6. Conclusions

In conclusion, biostimulants represent an emerging tool with great potential to promote more sustainable and resilient agricultural systems. The production of microalgae-based biostimulants can be structured in three key stages: (1) controlled microalgae cultivation, (2) extraction and characterization of bioactive compounds, and (3) application in agricultural systems (Figure 1). Current evidence indicates that the integration of microbial and non-microbial biostimulants improves soil functionality and long-term stability, as their effects tend to be cumulative rather than immediate. Furthermore, while biostimulants do not replace chemical fertilizers, they will play a complementary role by optimizing nutrient uptake, reducing soil degradation, and strengthening crop resilience to abiotic stress.

However, unregulated use or a lack of standardization in biostimulant formulations can lead to inconsistent results, interfere with agricultural management practices, and potentially alter the soil microbiota. The lack of clear regulatory frameworks also limits their comparative evaluation and commercial acceptance. Therefore, establishing robust regulations, validating mechanisms of action under diverse soil and climate conditions, and assessing long-term effects on soil health and productivity are essential steps for responsible implementation.

Overall, microalgae-derived biostimulants constitute a viable and sustainable strategy for improving agricultural productivity, provided their development and application are governed by technical and regulatory standards that guarantee their efficacy, safety, and environmental sustainability.

## Figures and Tables

**Table 1:** Microalgae-based biostimulants applied to agricultural crops.

Microalgae Strain	Growth Medium	Application (Plant/Seed)	Application Method	Effect	References
*Chlorella vulgaris* *Spirulina platensis*	N/A	Mung beans (*Vigna radiata* L.)	Foliar spraying	-Significant increase in root nodules and fresh weight of nodules in treated plant -Increase in the number of pods and seeds per treated plant	[30]
*Chlorella* (MACC-360)	Tris-acetate-phosphate with a pH 7.0 at 25 °C	*Medicago truncatula*	Soil drench method	-Increase in overall plant length by 11% -Up to 36% more flowers per plant -Total fresh weight increase by 36% Carotenoids increase by 31%	[31]
*Arthrospira platensis* (MS001) *Dunaleilla salina* (MS002) *Porphyridium* sp. (MS099)	Zarrouk medium in pH 9.0 at 30 °C (*A. platensis*) Walne’s medium with pH 8.2 at 25 °C (*Porphyridium* sp., *D. salina*)	Tomato (*Solanum lycopersicum*)	Irrigation	-Up to 25.26% increase in shoot length -46.61% shoot dry weight -75% increase in number of nodes.	[32]
*Chlorella vulgaris* *Arthrospira platensis* *Tetradesmus dimorphus*	Zarrouk’s medium pH 9.0 at 35 °C (*A. platensis*) Modified navicula medium pH 7.0 at 25 °C (*C. vulgaris*, *T. dimorphus*)	Beans (*Phaseolus vulgaris*)	Foliar Spraying	-Up to 27% increase in total plant length -37.3% increase in dry weight during vegetative state significant increase in total protein, total carbohydrates, total soluble sugars and polysaccharides across all treatments compared to control group.	[33]
Chlorococcum sp. *Micractinium pusillum* *Scenedesmus* sp. *Chlorella* sp.	Tris-acetate-phosphate pH 7.0 at 23 °C	Spinach (*Spinacia oleracea* L.)	Seed priming	-Up to 1.7 times more germinating seed by day 5 compared to control -3-fold increase in green cotyledon emergence by day 6 compared to water control -2.1 fold increase in total biomass by day 9 compared to control	[34]

**Table 2:** Applications of microalgae and microorganisms in plant biostimulation.

Microalgae/Bacteria	Plant Studied	Type of Compound Applied	Main Effects Observed	References
*Arthrospira* sp., *Scenedesmus* sp.	Petunia × hybrida	Hydrolysates with phytohormones (foliar application)	Accelerated growth, reduced flowering time, increased development of roots, leaves and shoots	[85]
*Desmodesmus subspicatus*	*Solanum lycopersicum* (tomato)	Extracts with glycosides and zeatin (cytokinin)	Increased germination and root length; 1.5 g/L increased hypocotyl volume.	[86]
*Arthrospira platensis*, *Dunaliella salina*, *Porphyridium* sp.	*Solanum lycopersicum* (tomato)	Polysaccharides (extracts)	Larger shoot size and root dry weight; *Porphyridium* (1 mg/mL) with greater biochemical and enzymatic effect	[32]
*Tetradesmus obliquus*	Garden cress, mung beans, cucumber	Biomass (0.2 g/L)	Better germination and growth; more efficient biomass than supernatant; supply of macro and micronutrients	[77]
*Rhizobium* (symbiotic bacteria)	Legumes	Association symbiotic (nodules)	Nitrogen fixation, ammonia supply to the plant, metabolite exchange (e.g., sucrose)	[87]
Microbial consortium (not just microalgae)	Wheat (*Triticum aestivum*)	Organic fertilizer + microbial consortium (20% less chemical fertilizer)	Increased biomass and yield in the field compared to chemical fertilization alone	[88]

**Table 3:** Examples of biostimulants that induce tolerance to abiotic stress in plants.

Biostimulant/Extract	Crop/Plant	Type of Stress	Effect Observed	Reference
*Chlorella vulgaris*, *Scenedesmus quadricauda*	Lettuce, beetroot sugar bowl	General	↑ PAL activity, synthesis of phenolic compounds and flavonoids	[56]
*Chlorella vulgaris* (foliar vs. root)	Lettuce	General	Faster enzymatic response by foliar application	[82]
Nanofertilizers (Nanoparticles in foliar application)	Several crops	General	Faster growth than soil application	[112]
*Ascophyllum nodosum* extract	Tomato	Drought	↑ tolerance to water stress, accumulation of proline and protective proteins	[100]
*Chlorella vulgaris*	Broccoli	Drought	↑ absorption of nutrients, secondary metabolites and antioxidant defense	[113]
Mixture: *Dunaliella salina* + *Chlorella ellipsoidea* + *Arthrospira maxima* + *Aphanothece* sp. (5%)	Tomato	Salinity	↑ tolerance, nutrient absorption and growth	[81]
Exopolysaccharides of *Dunaliella salina*	Tomato	Salinity	↑ tolerance, biostimulant effect of polysaccharides	[114]
*C. vulgaris* + bacteria in consortium	Lettuce	Thermal	↑ yields, ↑antioxidant activity, ↑carotenoid levels	[115]

The arrow ↑ means that there was an increase in the observed effect.

## Data Availability

The original contributions presented in the study are included in the article, further inquiries can be directed to the corresponding author.
